# Changing Trends of Imaging in Angle Closure Evaluation

**DOI:** 10.5402/2012/597124

**Published:** 2012-05-13

**Authors:** Syril Dorairaj, James C. Tsai, Tomas M. Grippo

**Affiliations:** ^1^Hamilton Glaucoma Center, Shiley Eye Center and Department of Ophthalmology, University of California, La Jolla, CA 92093, USA; ^2^Department of Ophthalmology and Visual Science, Yale University School of Medicine, New Haven, CT 06510, USA

## Abstract

Primary angle closure glaucoma (PACG) is a significant cause of visual disability worldwide. It predominantly affects the Eastern and South Asian population of the world. Early detection of anatomically narrow angles is important, and the subsequent prevention of visual loss from PACG depends on an accurate assessment of the anterior chamber angle (ACA). Gonioscopy has given way to modern day imaging technologies such as ultrasound biomicroscopy (UBM) and more recently, anterior segment optical coherence tomography (AS-OCT). Ultrasound biomicroscopy provides objective, high-resolution images of anterior segment anatomy, including the cornea, iris, anterior chamber, anterior chamber angle, and ciliary body. Optical coherence tomography (OCT) is a noncontact optical signal acquisition and processing device that provides magnified, high-resolution cross-sectional images of ocular tissues. Recent technological advances towards three-dimensional visualization broadened the scope of AS-OCT in ophthalmologic evaluation. Optical coherence tomography systems use low-coherence, near-infrared light to provide detailed images of anterior segment structures at resolutions exceeding that of UBM. This paper summarizes the clinical application of UBM and OCT for assessment of anterior segment in glaucoma.

## 1. Introduction

Primary angle closure glaucoma (PACG) is a leading cause of blindness worldwide [1]. It is potentially preventable if diagnosed early in the course of the disease, before irreversible damage has occurred to the optic nerve or trabecular meshwork (TM). Primary angle closure glaucoma comprises about 10% of glaucoma patients in the USA, and its prevalence varies amongst ethnic and racial groups [2]. Narrow angles are found in about 2% of Caucasians, with 0.1% having acute angle closure glaucoma [3–5]. The ethnic group in which PACG is most common is Eskimos [6]. Angle-closure is less common in blacks but more likely to develop chronic ACG when they do develop the disease [7–9]. Asians are prone to chronic angle-closure and often do not reach clinical attention until severe ocular damage has already occurred. In Asians, specifically, the incidence of angle-closure glaucoma outnumbers open-angle glaucoma [10]. In all racial and ethnic populations, ACG is found 3-4 times more often in females than males [11]. Finally, ACG is most prevalent in hyperopic and elderly patients, peaking between ages 55 and 70, since the anterior chamber depth and volume decrease with age due to nuclear sclerosis and in patients with a family history of angle-closure glaucoma [11].

Angle-closure glaucoma is three times as likely as open angle glaucoma (OAG) to cause blindness. Angle-closure glaucoma studies conducted in Asian countries estimate 4.3 million blind from ACG and 3.3 million from OAG and that bilateral blindness affects fewer than 10% of those with OAG but 25–30% of ACG sufferers [5, 12]. Needless to say, ACG is a worldwide problem to which attention should be paid with special regards to prevention and diagnosis.

Anterior segment pathologies, resulting from anatomic, structural, or mechanical abnormalities which cause apposition of the iris to the trabecular meshwork can contribute to angle-closure and thus the risk of progressive trabecular damage, elevated intraocular pressure (IOP), peripheral anterior synechiae (PAS), and acute angle-closure. Primary angle-closure glaucoma can, like other types of glaucoma, ultimately result in blindness if not diagnosed and treated timely.

 The pathophysiology of angle closure can be divided into primary and secondary causes. Primary angle closure is known as pupillary block, and it accounts for more than 90% of cases. In pupillary block, the flow of aqueous from the posterior chamber, where it is produced by nonpigmented ciliary epithelium, to the anterior chamber is limited because of resistance to aqueous flow through the pupil in the region of iridolenticular contact. This limitation of flow creates an increased pressure gradient between the anterior and posterior chambers, which in turn forces the iris anteriorly and causes anterior iris bowing, narrowing of the angle, and acute/chronic or acute on chronic iridotrabecular apposition or angle closure glaucoma. In relative pupillary block, all the anatomical structures are usually normal. Secondary causes of angle closure, by contrast, can result from structural or anatomic abnormalities in the anterior or posterior segments, such as plateau iris configuration, lens subluxation, or malignant glaucoma (ciliary block or aqueous misdirection).

Clinically, the gold standard for diagnosis of narrow angles is dark-room gonioscopy, in which the iridocorneal angle and aqueous outflow through the trabecular meshwork can be assessed; however, this technique is subjective, and there are currently no standards related to gonioscopy to determine which angles require treatment.

**Figure 1:**
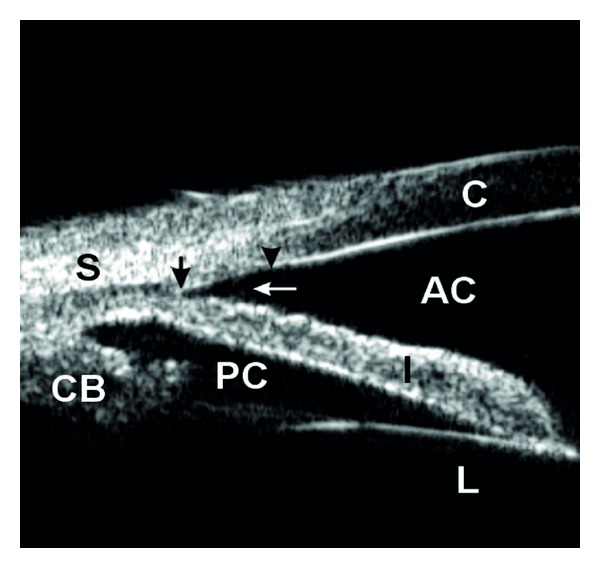
Ultrasound biomicroscopy image showing normal angle structures. S: sclera; CB: ciliary body, PC: posterior chamber, AC: anterior chamber, L: lens, C: cornea. Dark arrows delineate the trabecular meshwork from the scleral spur towards schwalbe's line while the white arrow signals points towards an open angle.

**Figure 2:**
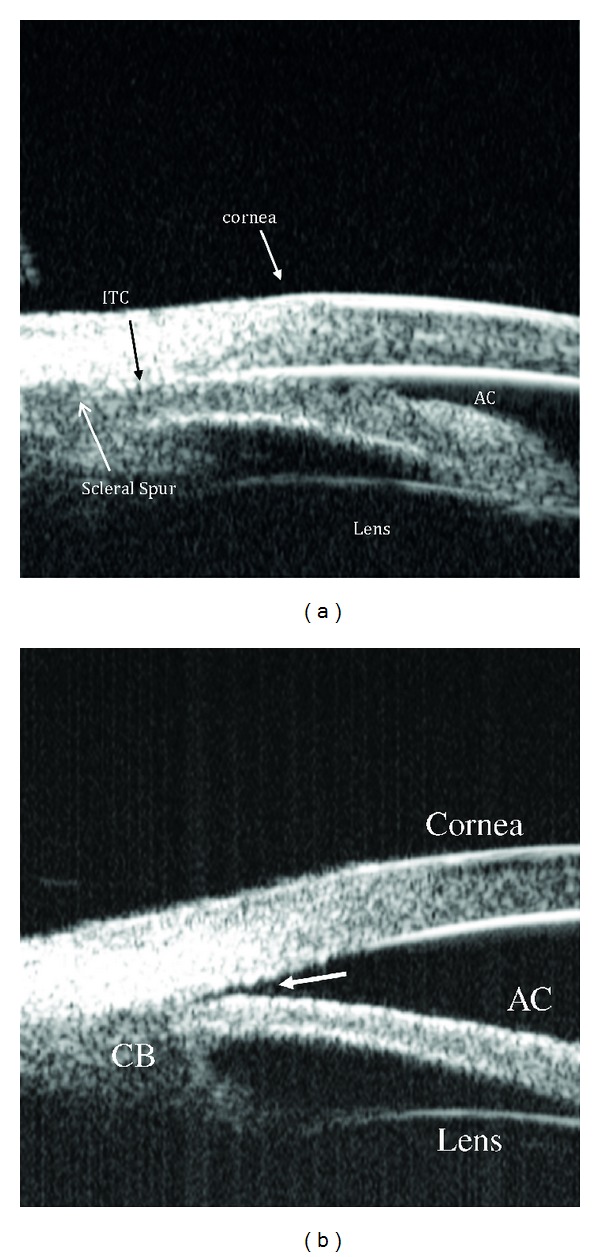
UBM image showing relative pupillary block with bowing of the iris anteriorly prior to laser iridotomy.

**Figure 3:**
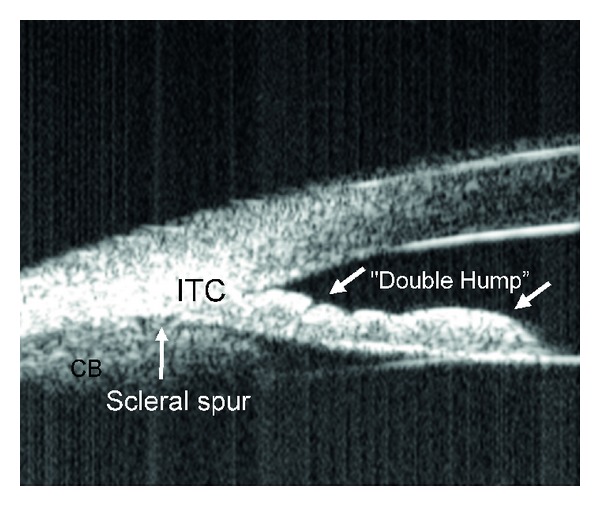
Ultrasound biomicroscopy image showing plateau iris with the classic double-hump sign. Contrary to angle closure on the basis of relative pupillary block, where indentation gonioscopy results in deepening of the peripheral anterior chamber, in plateau iris the iris contour follows the lens, dips posteriorly, then rises anteriorly before reaching the angle recess. The iris root remains angulated forward with a deepening of the anterior chamber confined to the region of the central iris. In this figure iridotrabecular contact (ITC) can be appreciated.

**Figure 4:**
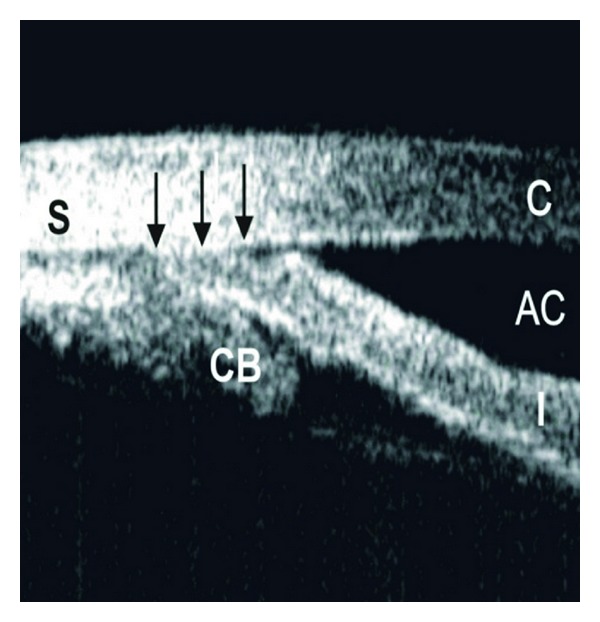
Ultrasound biomicroscopy image showing peripheral anterior synechia (PAS). S: sclera; CB: ciliary body, AC: anterior chamber, I: iris, C: cornea. Dark arrows delineate the PAS.

**Figure 5:**
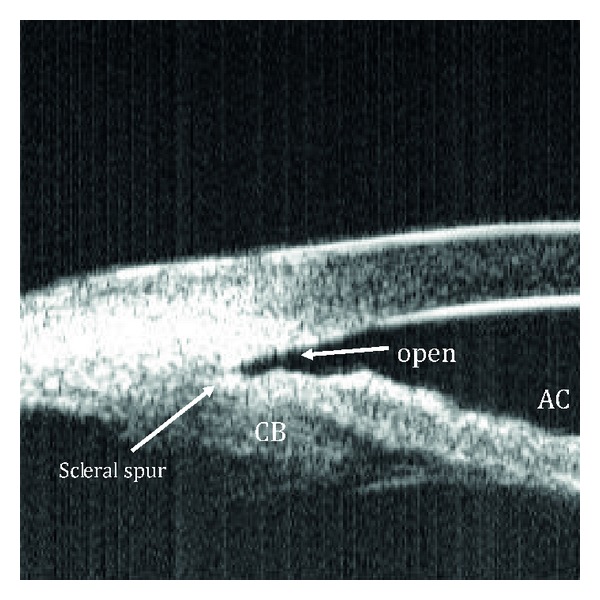
Ultrasound biomicroscopy image of an eye with plateau iris configuration status post-argon laser peripheral iridoplasty (PICP). An open angle and no ITC can be appreciated.

**Figure 6:**
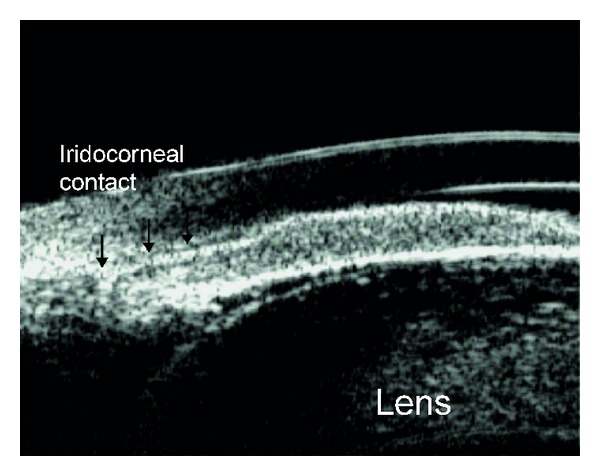
Ultrasound biomicroscopy image showing angle closure consequent of phacomorphic causes. A large lens is pushing iris anteriorly causing angle closure.

**Figure 7:**
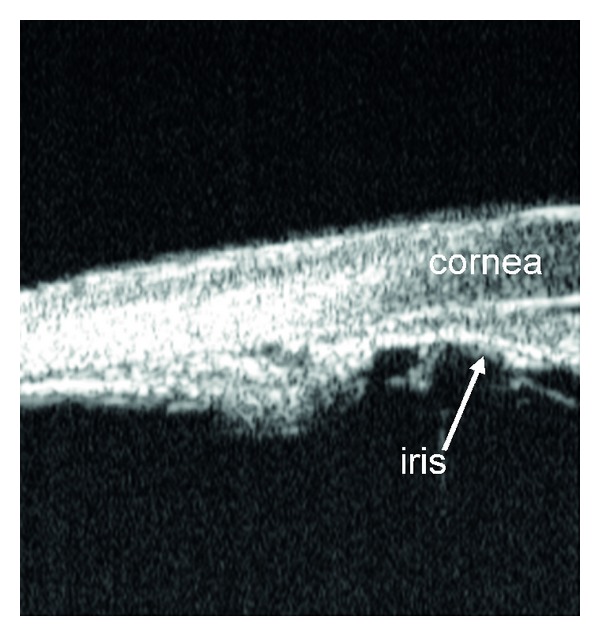
Ultrasound biomicroscopy image showing anteriorly rotated ciliary body in aqueous misdirection/ciliaryblock/malignant glaucoma.

Qualitative studies of the anterior segment structures can provide only limited information for diagnosis and subsequently, fail to provide a single, worldwide standard of care for narrow angles. Ideally, quantitative studies of the anterior chamber and the relationships of structures therein could provide objective measurements, which will standardize the anterior chamber (AC) parameters requiring interventions and treatments. The best way to achieve these quantitative measurements is through anterior chamber imaging devices. Ultrasound Biomicroscopy (UBM) has been used for this very purpose for more than 15 years [13–20]. UBM provides objective, high-resolution images of anterior segment anatomy, with tissue resolution of approximately 50 microns and penetration depth of 5 mm, providing a useful diagnostic tool for narrow angles and other anterior chamber pathologies. UBM is capable of imaging the cornea, iris, anterior chamber, anterior chamber angle, posterior chamber, and ciliary body (please see Figure 1). All UBM images shown in this manuscript were taken with a UBM model P40 from Paradigm Medical Industries, Salk Lake City, Utah. The various forms of angle closure glaucoma, such as pupillary block and plateau iris configuration can be differentiated using a UBM (Please see Figures 2(a), 2(b) and 3). Please see Figures 4, 5, 6, 7 for UBM image examples of peripheral anterior synechia, plateau iris configuration after argon laser peripheral iridoplasty (ALPI), phacomorphic angle closure, and malignant glaucoma.

Imaging technologies have proven extremely useful for explaining the nature of various pathologies and in determining a rationale for treatment in patients who may be confused concerning open-angle and angle-closure glaucoma and the laser treatment modality which best suits their condition.

In a UBM image, the scleral spur can be seen as the innermost point of the line separating the ciliary body and the sclera at its point of contact with the anterior chamber (please see Figure 1). The trabecular meshwork is located directly anterior to this structure and posterior to Schwalbe's line, which is the most peripheral portion of Descemet's membrane (please see Figure 1). Thus, the essential structures for the diagnosis of angle-closure glaucoma are clearly visible in a UBM image [21].

Despite the advantages of UBM, there are some disadvantages as well [22–24]. First, this procedure can be uncomfortable for the patient, requiring placement of an eyecup between the lids while lying in the supine position. The eyecup is filled with saline, and a 35–50 MHz transducer is placed in the saline as the patient looks down, right, up, and left to record the superior, nasal, inferior, and temporal angles. The exam occurs once in room lighting and once with the lights turned off, known as the light and dark provocative test, to look for appositional closure and angle occludability. Many patients have difficulty tolerating this procedure. Imaging with the UBM carries some risks for the patient, such as scratching the cornea and requires a skilled and trained operator. In addition, positional changes, such as the supine position used during UBM testing, may alter relationships between chamber structures, particularly in eyes with narrow angles. In the supine position, at least in some patients, the lens might move posteriorly, thus missing some occludable angles during UBM testing.

 The risks of performing UBM, the requirement for a skilled operator, and the incomplete or nonideal information provided by the supine position leave room for improvement in anterior segment imaging. The next generation of imaging would provide images taken in the seated position, preferably without any contact, and, with less invasiveness, less risk and less discomfort for the patient (please see Table 1 for details of UBM as well as ASOCT devices available in the market).

**Figure 8:**
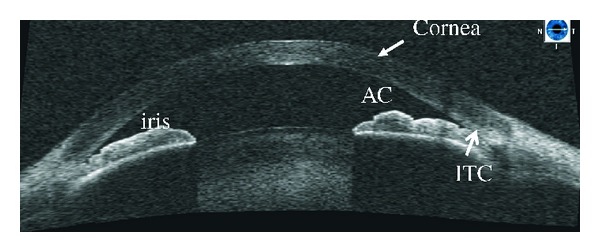
Anterior segment optical coherence tomography image showing relative pupillary block. AC: anterior chamber; ITC: iridotrabecular contact.

**Figure 9:**
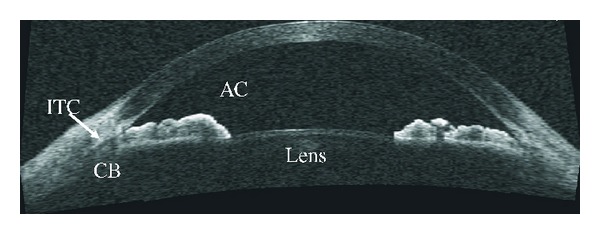
Anterior segment optical coherence tomography image showing plateau iris configuration. CB: ciliary body, AC: anterior chamber, ITC: iridotrabecular contact.

**Figure 10:**
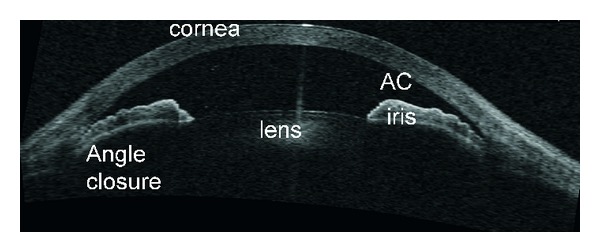
Anterior segment optical coherence tomography image showing angle closure consequent of phacomorphic causes.

**Figure 11:**
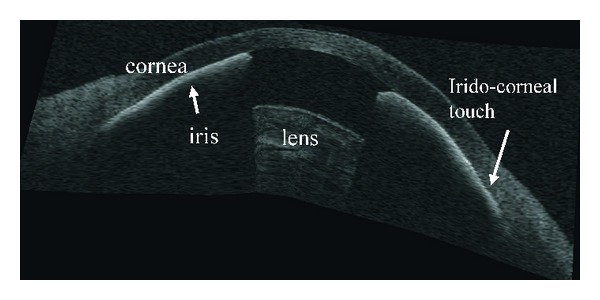
Anterior segment optical coherence tomography image showing iridotrabecular and iridocorneal contact in a case of aqueous misdirection/ciliaryblock/malignant glaucoma.

With new anterior segment optical coherence tomography (ASOCT) imaging techniques, detailed spatial relationships of the anterior segment structures can be visualized and objective anterior chamber angle (ACA) measurements can be performed in a noncontact manner (please see Figure 8). In addition, the use of infrared laser and real time eye position monitor during examination permits the precise capture of angle morphology in the dark. With higher scan speed, slit lamp optical coherence tomography (SLOCT) has the potential to provide valuable quantitative and spatial information regarding dynamic changes of the angle configuration, which cannot be provided by standard gonioscopy and UBM. SLOCT provides information that is comparable to an intermediate standard between ASOCT and UBM in terms of ACA measurement [25]. Please see Figures 8, 9, 10, 11 for examples of AS-OCT images of relative pupillary block, plateau iris, phacomorphic glaucoma, and malignant glaucoma. All ASOCT images shown in this paper were taken with a slit-lamp-adapted anterior segment OCT from Heidelberg Engineering, Heidelberg, Germany.

Enhancing the clinical applicability of ASOCT, Prata et al., described a novel dynamic technique to differentiate appositional from synechial angle closure and to understand the underlying mechanisms of angle closure using indentation ASOCT [26]. Identification of the causes of angle closure is of utmost importance, as each case may have a different course therefore requiring a different treatment approach. Differentiation of appositional and synechial angle closure in eyes with iridotrabecular contact during indention ASOCT adds to the clinical utility of ASOCT in the evaluation of patients with angle closure.

Asrani et al. recently reported the successful visualization of details of anterior chamber drainage angle (Schlemm's canal, trabecular meshwork and configuration details of the iris with respect to the angle) using a swept source Fourier-domain OCT system [27].

## 2. The Reliability of ACA Measurement

The scleral spur is a protrusion of sclera anchoring the trabecular meshwork anteriorly and the longitudinal muscle of the ciliary body posteriorly. It represents an anatomical landmark for the trabecular meshwork which is located approximately 250 to 500 *μ*m anterior to the scleral spur along the angle wall. Because of the different tissue reflectivity between the sclera and cornea, the scleral spur can be visualized in UBM or OCT as a distinct anatomical landmark for measurement of the ACA [21, 28, 29]. Because most of the important parameters for ACA quantitative measurements are based on the identification of the point of scleral spur, reliable documentation of the angle dimensions is therefore dependent on precise and repeatable localization of the scleral spur. Sakata et al. found that, on the same Visante OCT images, the intraobserver agreement in detecting the scleral spur (132 quadrants) was moderate to substantial with *κ* = 0.65 [30]. They also reported that, in the assessment of the exact scleral spur location, the distance between the scleral spur localized in the same image across 2 sessions was within 10 *μ*m in 83% of the 78 quadrants assessed and within 20 *μ*m in 90%. The location of the scleral spur on ASOCT images was less detectable in quadrants with a closed angle on gonioscopy (odds ratio = 0.54, *P* = 0.02) and also in images obtained in the superior and inferior compared with the nasal and temporal quadrants (64%, 67%, 75%, and 80%, respectively; *P* < 0.001). Using the same images for measurement, the intraobserver coefficient of variation (CVw) of angle opening distance (AOD) ranged between 4.9% and 7.8% by ASOCT and was up to 16.97% by UBM (*P* < 0.001) which indirectly indicates that identification of scleral spurs might be more repeatable by ASOCT than UBM [25–31].

The differences in anterior chamber angle measurements in different lighting conditions have been investigated with UBM and ASOCT [32]. Leung et al. also described the dynamic ACA changes induced by dark-light changes by Visante OCT through real-time video recording [33]. They found that the AOD and trabecular iris space area (TISA) decreased linearly with increasing pupil size in most cases (85.5% in AOD and 90.9% in TISA). It was estimated that for each mm change in pupil size, there was an average of 94 *μ*m change in the AOD and 0.035 mm^2^ change in the TISA. Although significant differences of angle measurements were found between light and dark conditions, good repeatability and reproducibility were achieved as long as the lighting condition had been standardized by ASOCT [33, 34]. The intersession CVw for angle measurements by Visante OCT was less than that of UBM (Visante OCT: 6~11%, UBM: 16~18%) [31, 33].

Significant correlations were found among ACA measurements by ASOCT, gonioscopy, and UBM [29, 35]. In general, the correlation in detecting a closed ACA quadrant using ASOCT and gonioscopy was fair with a *κ* of 0.4. But ASOCT tended to detect more closed angles than gonioscopy, particularly in the superior and inferior quadrants [30]. There was no significant difference in angle measurements between ASOCT and UBM in either nasal or temporal quadrants, but a significant higher AOD measurement was observed by ASOCT in the superior and inferior angles compared with UBM [29, 36]. Of note, although slit-lamp OCT (SLOCT) and Visante OCT generally had no significant difference in angle measurements, the two available ASOCT models had poor correlations in ACA measurement despite comparable pupil diameters obtained, with the spans of 95% limits of agreement (LOA) of the nasal/temporal angle measurements between them being 437 *μ*m/531 *μ*m, 0.174 mm^2^/0.186 mm^2^, and 25.3°/28.0° for AOD, TISA, and trabecular-iris angle (TIA), respectively [24]. The poor correlation is likely related to differences in the choice of refractive indexes in the calculation of anterior segment dimensions, algorithms for image dewarping, the exact scan locations, and the state of accommodation.

Furthermore, UBM offers a better view of the ciliary body, which is rarely visible during ASOCT, since attenuated light from the overlying sclera obscures the view of the ciliary body. However, recent studies have confirmed the ability of ASOCT to evaluate and confirm a clinical suspicion of plateau iris configuration and syndrome [37].

Finally, different factors can influence the appearance of the angle, including background illumination, blinking, patient posture, contact with the eye, and image processing software.

In most cases the software to analyze OCT images is based on manual labeling of the scleral spur, cornea, and iris which is not only a tedious process but sometimes not possible to accomplish as in about 20–30% of the cases; these landmarks cannot be identified. Sakata et al. found that the sclera spur could not be detected in approximately 30% of the ACA quadrants, this problem being worse in the superior and inferior quadrants [30]. In order to overcome this limitation Jing et al. suggested a new algorithm capable of automatically detecting Schwalbe's line in HD-OCT scans [38].

Clinically, ASOCT has been applied to the observation of ACA change after glaucoma surgeries, such as laser peripheral iridotomy (LPI), argon laser peripheral iridoplasty (ALPI), trabeculectomy combined with cataract extraction, and intraocular lens implantation.

In a retrospective study involving 71 Caucasian eyes, Ang and Wells compared AS-OCT parameters before and after laser iridotomy [39]. The authors found that this procedure resulted in significant angle widening as shown on increased TIA, AOD, TISA, and iris profile flattening. In an Asian population of 46 patients, Lee et al. [40] found that when assessed by measurement variability criteria, the percentage of eyes that showed no significant change in ACA parameters ranged from 23.9% to 45.7% after LPI. In 15 patients with primary angle closure Lei et al. [41] found that after LPI the peripheral anterior chamber depth and anterior chamber volume increased as well as the central anterior chamber depth increased as seen on AS-OCT.

In most of the priory reported studies where LPI was done, the authors found a proportion of patients in whom the angle did not widen but did not specify the possible etiologies for it, and this is consistent with the limitation of current OCT technologies to evaluate the structures behind the iris like an anterior insertion of the ciliary body causing plateau iris configuration/syndrome. Despite this limitation in a small case series some authors have suggested the presence of signs that are suspicious for plateau iris syndrome in AS-OCT [42]. Both OCT and UBM showed excellent performance in identifying eyes with plateau iris. The UBM confirmed the plateau iris diagnosis by showing the iris root indentation caused by the ciliary body. The OCT can detect indirect signs of plateau iris syndrome after iridotomy [42].

## 3. Summary and Conclusions

It is envisioned that the new anterior segment imaging devices would have as significant impact as the new posterior segment imaging devices. The new imaging devices do not aim to replace conventional slit-lamp biomicroscopy. They would act to supplement and augment clinical practice and become invaluable tools for ophthalmic research. The major advantages of the newer devices are the noncontact nature of examination, high scan speed, good repeatability and reproducibility for quantitative and qualitative measurements, and cross-sectional visualization of anterior segment structures. Since ASOCT can visualize the entire anterior chamber, all the essential parameters for detection of angle closure/narrow angle can be examined in a single scan. The ASOCT would become an essential tool for screening PAC, making screening programs for PACG more feasible and less doctor dependent. The application of ASOCT has led to a better understanding of anterior segment diseases. It can now be readily quantified making longitudinal followups and assessments possible. The outcome of treatment can be monitored without discomfort or risks of inflammation. The new devices may improve our understanding of current limitations of surgery. The potential clinical applications of these methods are only starting to be explored and the range of information they may yield has yet to be determined. Therefore, the use of the newer anterior segment imaging devices could well be the start of a new era for ophthalmic diagnosis.

## Figures and Tables

**Table 1:** Presents the details of UBM as well as ASOCT devices available in the market.

	UBM (50 MHz)	Stratus-OCT	Visante-OCT (TD-OCT)	RTVue (FD-OCT)	Cirrus high-definition OCT (HD-OCT)
Light source	Ultrasound	Super luminescent Diode 820 nm	Super luminescent Diode 1310 nm	Super luminescent Diode 840 nm	Super luminescent Diode 840 nm
Scan size	Up to 7 mm tissue depth	6 mm (width) × 2 mm (depth)	16 mm × 6 mm	2 mm × 2 mm (CAM-S) OR 6 mm × 2 mm (CAM-L)	3 mm × 1 mm
Scans rate (A-scans/second)	1000	400	2,000	26,000	27,000
Axial resolution	30 *μ*m	10 *μ*m	18 *μ*m	5 *μ*m	5 *μ*m
